# Supplementary material for: Perceived Gap of Age-Friendliness among Community-Dwelling Older Adults: Findings from Malaysia, a Middle-Income Country
Source: Int J Environ Res Public Health. 2022 Jun 11;19(12):7171. doi: 10.3390/ijerph19127171 (PMC9223156; doi:10.3390/ijerph19127171)
Supplement: Supplementary file 1 [file ijerph-19-07171-s001.zip › ijerph-1694119-supplementary.pdf]

Supplementary Table S1: Availability and importance of the elements in eight domains of an aged-friendly city in Ipoh city's perspectives

		**Availability**							**Importance**						
		**Yes**		**No**		**Not sure**		**Total**	**Yes**		**No**		**Not sure**		**Total**
**Domain**	**Elements**	**n**	**%**	**n**	**%**	**n**	**%**	**n**	**n**	**%**	**n**	**%**	**n**	**%**	**n**
**Housing**	Homes that are equipped with features such as a no-step entry, wider doorways, first floor bedroom and bath, grab bars in bathrooms	559	52.7	284	26.8	217	20.5	1060	910	86.0	80	7.6	68	6.4	1058
	Affordable housing options for senior people	203	19.2	617	58.2	240	22.6	1060	804	75.9	154	14.5	101	9.5	1059
	Financial assistance for home modification/ purchasing	128	12.1	621	58.6	310	29.3	1059	737	69.8	195	18.5	124	11.7	1056
**Outdoor spaces and buildings**	Accessibility of the following to older people:														
	Park and recreational areas	801	75.6	127	12.0	131	12.4	1059	972	92.0	20	1.9	65	6.2	1057
	Public building and facilities (e.g. hospital, post office, bank, offices, telephone booth, others)	930	87.8	85	8.0	44	4.2	1059	1008	95.4	14	1.3	35	3.3	1057
	Public rest rooms accessible to older people of different physical abilities	614	58.0	184	17.4	261	24.7	1059	939	88.9	20	1.9	97	9.2	1056
	Well-maintained parks, public building and facilities	798	75.4	171	16.2	90	8.5	1059	1011	95.7	11	1.0	35	3.3	1057
	Neighborhood watch programs (eg: *RELA, Rukun Tetangga*)	542	51.2	413	39.0	104	9.8	1059	956	90.4	33	3.1	68	6.4	1057
**Transportation and streets**	Accessible and convenient public transportation	452	42.8	365	34.5	240	22.7	1057	893	84.6	28	2.7	135	12.8	1056

	Affordable public transportation	442	41.7	104	9.8	513	48.4	1059	891	84.3	20	1.9	146	13.8	1057
	Public transport provides access to destinations like hospital, clinics, parks, shopping centers, banks and other key destination.	461	43.5	102	9.6	496	46.8	1059	903	85.4	18	1.7	136	12.9	1057
	Easy to read traffic signs (e.g. appropriate size, color and font)	846	79.9	86	8.1	127	12.0	1059	993	93.9	9	0.9	56	5.3	1058
	Priority parking bays for elderly	130	12.3	775	73.2	154	14.5	1059	954	90.2	32	3.0	72	6.8	1058
	Audio / visual pedestrian crossings	408	38.5	463	43.7	188	17.8	1059	967	91.5	15	1.4	75	7.1	1057
**Health and wellness**	Health and wellness programs and classes in areas such as nutrition, smoking cessation, and weight control	369	34.8	381	36.0	309	29.2	1059	865	81.8	63	6.0	130	12.3	1058
	Conveniently located health facilities	971	91.7	67	6.3	21	2.0	1059	1040	98.2	4	0.4	15	1.4	1059
	Home visit services by healthcare professionals for senior patients	179	16.9	637	60.2	243	23.0	1059	951	89.9	27	2.6	80	7.6	1058
	Nursing home for older people	698	65.9	250	23.6	111	10.5	1059	859	81.2	112	10.6	87	8.2	1058
	A variety of health care professionals including specialists	710	67.0	171	16.2	178	16.8	1059	1026	96.8	5	0.5	29	2.7	1060
	Health care professionals who speak different languages	818	77.2	135	12.8	106	10.0	1059	1011	95.5	23	2.2	25	2.4	1059
	Respectful and helpful hospital and clinic staff	988	93.3	28	2.6	43	4.1	1059	1035	98.1	3	0.3	17	1.6	1055

**Social participation, inclusion and education opportunities**	Privilege for senior citizens for entertainment (e.g. discount, special program, no queue, etc.)	298	28.2	406	38.4	354	33.5	1058	596	56.4	306	29.0	154	14.6	1056
	Conveniently located venues for entertainment	352	33.3	297	28.1	409	38.7	1058	449	42.5	414	39.2	194	18.4	1057
	A variety of cultural celebration (festivals, spiritual events, etc.) involving older adults in the multiracial populations	547	51.8	364	34.5	145	13.7	1056	811	76.8	147	13.9	98	9.3	1056
	Social clubs for books, gardening, crafts or hobbies	279	26.4	506	47.9	272	25.7	1057	501	47.3	376	35.5	182	17.2	1059
**Volunteering and civic engagement**	A range of volunteer activities to choose from for elderly	275	25.9	531	50.1	254	24.0	1060	492	46.5	408	38.5	159	15.0	1059
	Opportunities for older adults to participate in decision making bodies such as community councils or committees (e.g. giving expert opinion)	351	33.2	459	43.4	248	23.4	1058	529	50.1	377	35.7	151	14.3	1057
**Job opportunities**	A range of flexible job opportunities for older adults (e.g. part-time)	214	20.2	647	61.1	198	18.7	1059	579	54.7	389	36.7	91	8.6	1059
**Community and information**	Official, written information (e.g.: forms, brochures) adapted to the needs of seniors (e.g. Large font size)	699	65.9	246	23.2	115	10.9	1060	951	89.8	36	3.4	72	6.8	1059
	Telephone operator services adapted to the needs of seniors (e.g.: instructions are given slowly)	621	58.5	166	15.7	274	25.8	1061	893	84.4	26	2.5	139	13.1	1058
	Free access to computers and the Internet in public places such as the library, centers or government buildings	210	19.8	422	39.9	427	40.3	1059	646	61.2	213	20.2	197	18.7	1056
	Information (e.g. flyers, maps, others) that is available in a number of different languages	550	51.8	371	35.0	140	13.2	1061	893	85.1	72	6.9	84	8.0	1049

Supplementary Table S2: Gap score analysis of the elements in eight domains of an aged-friendly city in Ipoh city's perspectives

Domain	Elements	Valid n[a]	Score 0: No perceived gap		Score 1: Perceived gap		Score 2: Perceived gap		Mean Percent	
			n	%	n	%	n	%		
**Housing**	Homes that are equipped with features such as a no-step entry, wider doorways, first floor bedroom and bath, grab bars in bathrooms	990	630	63.6	117	11.8	243	24.5	36.4	55.4
	Affordable housing options for senior people	958	349	36.4	125	13.0	484	50.5	63.6	
	Financial assistance for home modification/ purchasing	932	314	33.7	157	16.8	461	49.5	66.3	
**Outdoor spaces and buildings**	Accessibility of the following to older people:	992	811	81.8	68	6.9	113	11.4	18.2	25.5
	Park and recreational areas	1022	929	90.9	15	1.5	78	7.6	9.1	
	Public building and facilities (e.g. hospital, post office, bank, offices, telephone booth, others)	963	625	64.9	168	17.4	170	17.7	35.1	
	Public rest rooms accessible to older people of different physical abilities	1022	800	78.3	55	5.4	167	16.3	21.7	
	Well-maintained parks, public building and facilities	989	561	56.7	53	5.4	375	37.9	43.3	
**Transportation and streets**	Accessible and convenient public transportation	919	471	51.3	108	11.8	340	37.0	48.7	50.7
	Affordable public transportation	911	456	50.1	360	39.5	95	10.4	49.9	
	Public transport provides access to destinations like hospital, clinics, parks, shopping centers, banks and other key destination.	921	470	51.0	357	38.8	94	10.2	49.0	
	Easy to read traffic signs (e.g. appropriate size, color and font)	1001	843	84.2	76	7.6	82	8.2	15.8	
	Priority parking bays for elderly	986	160	16.2	85	8.6	741	75.2	83.8	

	Audio / visual pedestrian crossings	982	421	42.9	118	12.0	443	45.1	57.1	
**Health and wellness**	Health and wellness programs and classes in areas such as nutrition, smoking cessation, and weight control	927	419	45.2	166	17.9	342	36.9	54.8	31.3
	Conveniently located health facilities	1044	968	92.7	14	1.3	62	5.9	7.3	
	Home visit services by healthcare professionals for senior patients	978	206	21.1	168	17.2	604	61.8	78.9	
	Nursing home for older people	971	754	77.7	53	5.5	164	16.9	22.3	
	A variety of health care professionals including specialists	1030	712	69.1	156	15.1	162	15.7	30.9	
	Health care professionals who speak different languages	1033	837	81.0	80	7.7	116	11.2	19.0	
	Respectful and helpful hospital and clinic staff	1039	980	94.3	31	3.0	28	2.7	5.7	
**Social participation, inclusion and education opportunities**	Privilege for senior citizens for entertainment (e.g. discount, special program, no queue, etc.)	902	588	65.2	91	10.1	223	24.7	34.8	27.9
	Conveniently located venues for entertainment	862	703	81.6	62	7.2	97	11.3	18.4	
	A variety of cultural celebration (festivals, spiritual events, etc.) involving older adults in the multiracial populations	955	670	70.2	29	3.0	256	26.8	29.8	
	Social clubs for books, gardening, crafts or hobbies	872	625	71.7	62	7.1	185	21.2	28.3	
**Volunteering and civic engagement**	A range of volunteer activities to choose from for elderly	900	665	73.9	66	7.3	169	18.8	26.1	23.7
	Opportunities for older adults to participate in decision making bodies such as community councils or committees (e.g. giving expert opinion)	905	712	78.7	57	6.3	136	15.0	21.3	
**Job opportunities**	A range of flexible job opportunities for older adults (e.g. part-time)	968	573	59.2	64	6.6	331	34.2	40.8	40.8
**Communication & information**	Official, written information (e.g.: forms, brochures) adapted to the needs of seniors (e.g. Large font size)	987	714	72.3	56	5.7	217	22.0	27.7	37.2

Telephone operator services adapted to the needs of seniors (e.g.: instructions are given slowly)	919	639	69.5	136	14.8	144	15.7	30.5
Free access to computers and the Internet in public places such as the library, centers or government buildings	861	410	47.6	159	18.5	292	33.9	52.4
Information (e.g. flyers, maps, others) that is available in a number of different languages	963	595	61.8	60	6.2	308	32.0	38.2

[a]*Responses were considered invalid if missing data or unsure importance*

Supplementary Table S3: Univariate binary logistic regressions assessing demographic characteristics associated with presence of perceived gap on the eight age-friendly domains

Domain	Housing	Outdoor spaces and buildings	Transportation	Health and wellness	Social participation	Volunteering and civic engagement	Job opportunities	Community & information
				Crude OR (95% CI)				
**Age group**								
**60-70**								
**71-80**	0.788 (0.577-1.075)	0.955 (0.722-1.263)	1.074 (0.689-1.675)	1.250 (0.845-1.850)	0.812 (0.615-1.072)	0.935 (0.690-1.268)	0.814 (0.610-1.085)	1.287 (0.948-1.749)
**81 and above**	0.450 (0.264-0.765)	0.876 (0.523-1.468)	0.735 (0.348-1.549)	0.781 (0.412-1.480)	0.824 (0.485-1.398)	0.603 (0.311-1.170)	0.632 (0.358-1.116)	0.923 (0.528-1.615)
**Gender**								
**Male**								
**Female**	0.880 (0.664-1.168)	1.031 (0.802-1.327)	0.709 (0.476-1.056)	1.116 (0.796-1.565)	0.999 (0.776-1.287)	0.937 (0.713-1.232)	0.719 (0.719-1.202)	1.119 (0.855-1.464)
**Location (city centre vs non-city centre)**								
**City-centre**								

**Non-city centre**	0.870 (0.657-1.154)	0.785 (0.610-1.011)	0.977 (0.660-1.445)	1.405 (0.998-1.977)	1.372 (1.065-1.768)	0.684 (0.520-0.901)	0.962 (0.745-1.244)	0.869 (0.664-1.138)
**Marital status**								
**Married**								
**Unmarried**	0.919 (0.635-1.330)	0.809 (0.585-1.119)	0.634 (0.398-1.010)	0.988 (0.634-1.539)	1.054 (0.754-1.472)	0.835 (0.572-1.220)	1.113 (0.796-1.555)	1.593 (1.085-2.340)
**Ethnicity**								
**Malay**								
**Chinese**	0.980 (0.679-1.415)	1.406 (1.009-1.957)	1.487 (0.887-2.492)	1.239 (0.795-1.931)	1.208 (0.867-1.682)	1.268 (0.897-1.792)	1.161 (0.831-1.623)	1.071 (0.758-1.514)
**Indian**	0.956 (0.689-1.327)	1.335 (0.998-1.788)	1.421 (0.906-2.227)	1.183 (0.802-1.744)	1.062 (0.793-1.424)	0.736 (0.531-1.020)	1.007 (0.747-1.358)	1.292 (0.944-1.768)
**Others**	2.075 (0.456-9.447)	1.381 (0.463-4.120)	2.117 (0.272-16.461)	2.800 (0.362-21.679)	1.242 (0.408-3.782)	0.840 (0.258-2.736)	1.079 (0.336-3.468)	3.104 (0.689-13.980)
**Education**								
**None**								
**Primary**	1.371 (0.726-2.587)	0.981 (0.534-1.800)	0.612 (0.180-2.074)	1.011 (0.430-2.374)	0.748 (0.395-1.417)	2.549 (1.102-5.895)	1.120 (0.579-2.166)	0.768 (0.374-1.577)
**Secondary**	1.502 (0.807-2.798)	1.086 (0.598-1.970)	0.495 (0.149-1.642)	0.863 (0.375-1.984)	0.784 (0.419-1.466)	2.588 (1.133-5.911)	1.431 (0.750-2.730)	0.719 (0.355-1.456)
**Tertiary**	2.501 (1.200-5.216)	1.089 (0.558-2.123)	0.576 (0.158-2.100)	0.775 (0.310-1.934)	0.774 (0.387-1.546)	3.516 (1.459-8.476)	2.233 (1.097-4.547)	0.915 (0.419-1.999)
**Living status**								
**Alone**								

**Not alone**	0.812 (0.449-1.468)	1.233 (0.755-2.015)	1.060 (0.494-2.274)	0.812 (0.395-1.668)	0.970 (0.594-1.583)	0.965 (0.571-1.633)	0.692 (0.421-1.138)	0.779 (0.442-1.372)
**Income**								
**Less than RM 2000**								
**RM2000-4800**	1.216 (0.745-1.984)	0.754 (0.495-1.149)	1.106 (0.557-2.195)	0.839 (0.482-1.458)	0.966 (0.636-1.465)	0.952 (0.593-1.527)	12.117 (1.370-3.270)	0.818 (0.525-1.276)
**More than RM4800**	4.968 (1.172-21.053)	1.235 (0.556-2.743)	1.658 (0.389-7.079)	1.087 (0.372-3.171)	3.497 (1.322-9.245)	2.256 (1.060-4.800)	4.653 (1.946-11.126)	2.637 (0.909-7.652)
**Duration of stay**								
**6 months-10 years**								
**11-30 years**	1.299 (0.587-2.874)	1.500 (0.785-2.866)	1.912 (0.788-4.638)	2.984 (1.351-6.593)	0.992 (0.520-1.893)	0.765 (0.377-1.553)	0.873 (0.449-1.697)	0.672 (0.319-1.417)
**31-60 years**	1.024 (0.478-2.195)	1.558 (0.830-2.924)	1.564 (0.675-3.623)	1.430 (0.698-2.929)	1.129 (0.602-2.116)	0.754 (0.378-1.504)	1.045 (0.549-1.992)	0.773 (0.373-1.604)
**More than 60 years**	0.762 (0.364-1.595)	1.954 (1.058-3.609)	2.348 (1.026-5.375)	2.367 (1.166-4.805)	1.452 (0.787-2.680)	0.479 (0.243-0.942)	0.627 (0.334-1.176)	0.900 (0.441-1.836)
**Health condition**								
**Healthy**								
**Active with diseases**	1.053 (0.740-1.500)	0.879 (0.642-1.203)	1.222 (0.769-1.944)	0.826 (0.534-1.278)	0.756 (0.555-1.030)	0.921 (0.661-1.285)	0.789 (0.573-1.084)	0.874 (0.623-1.227)

**Inactive**	0.448 (0.234-0.859)	0.913 (0.481-1.734)	2.154 (0.626-7.417)	0.701 (0.309-1.590)	0.419 (0.226-0.778)	0.427 (0.180-1.017)	0.657 (0.335-1.287)	0.916 (0.438-1.915)
**Possess health care insurance**								
**Yes**								
**No**	0.758 (0.565-1.018)	0.897 (0.691-1.164)	0.814 (0.539-1.231)	0.756 (0.528-1.084)	0.789 (0.607-1.024)	0.963 (0.729-1.274)	0.828 (0.637-1.078)	0.969 (0.734-1.278)
**Physical exercise**								
**Never**								
**1-4 times per week**	1.099 (0.760-1.591)	1.056 (0.748-1.489)	1.192 (0.706-2.011)	1.033 (0.652-1.636)	1.281 (0.907-1.810)	1.226 (0.832-1.808)	0.950 (0.670-1.347)	0.914 (0.633-1.319)
**5-7 times per week**	1.754 (1.166-2.638)	1.222 (0.849-1.760)	1.149 (0.663-1.993)	1.100 (0.676-1.793)	1.226 (0.851-1.768)	1.322 (0.880-1.986)	0.797 (0.550-1.156)	1.288 (0.866-1.914)
**Internet access**								
**No**								
**Yes**	1.733 (1.301-2.307)	1.085 (0.843-1.395)	1.305 (0.879-1.937)	1.344 (0.956-1.891)	1.344 (1.044-1.732)	1.781 (1.348-2.353)	1.739 (1.342-2.253)	1.323 (1.010-1.734)
**Employment status**								
**Employed**								
**Unemployed**	0.999 (0.694-1.437)	1.048 (0.759-1.448)	1.393 (0.871-2.228)	1.307 (0.865-1.975)	0.911 (0.656-1.266)	0.752 (0.534-1.057)	0.605 (0.438-0.834)	1.152 (0.819-1.621)

**Intention to continue working**								
**Yes**								
**No**	0.874 (0.618-1.236)	1.035 (0.763-1.403)	1.184 (0.747-1.876)	1.208 (0.813-1.794)	0.932 (0.685-1.270)	0.610 (0.444-0.838)	0.461 (0.340-0.626)	1.042 (0.753-1.442)

CI, confidence interval; OR, odd ratio.

QUESTIONNAIRE

ZONE: __________ LOCATION: ____________________________________ DATA COLLECTOR (CODE) : _____________

Subject ID: _____________ Gender: F ☐ M ☐

ABOUT YOU

1. **What is your date of birth (DD/MM/YYYY)?** ☐☐ / ☐☐ / ☐☐☐☐
2. **What is your current marital status?**
   - ❍ Married
   - ❍ Widowed
   - ❍ Divorced / separated
   - ❍ Bachelor / never married
3. **What is your ethnicity?**
   - ❍ Malay
   - ❍ Indian
   - ❍ Chinese
   - ❍ Others, please specify: ______________________
4. **Besides yourself, do you have any of the following people living in your household currently?**
   - ❍ None
   - ❍ Other relatives
   - ❍ Spouse only
   - ❍ Your friend
   - ❍ Family members (spouse / children / grandchildren)
   - ❍ Others, please specify: ___________________
5. **What is your highest education (no education / primary / secondary / tertiary / religious education /etc.?**

____

BASELINE INFORMATION ON THE 8 DOMAINS FOR AGE-FRIENDLY CITY IN IPOH

6. How would you rate Ipoh as a place for senior citizens (age > 60 years) to live?

❍ Good ❍ Moderate ❍ Poor

7. Are you a local resident in Ipoh?

❍ Yes Please specify long have you lived in Ipoh: __________ (years) ___________ (months)

❍ No

8. How important is it for you to remain staying in Ipoh?

❍ Important ❍ Not important ❍ Not sure

9. How important is it for you to be able to live independently in your own home as you age?

❍ Important ❍ Not important ❍ Not sure

10. Do you think you will need to make the following types of modifications or improvements to your home to enable you to stay there as you age?

		Yes	No	Not sure	Not relevant
a.	Easier access into or within your home such as a ramp, chairlift or elevator, or wider doorways	❍	❍	❍	❍
b.	Bathroom modifications such as grab bars, handrails, a higher toilet or non-slip tiles	❍	❍	❍	❍
c.	Putting a bedroom, bathroom and kitchen on the first floor	❍	❍	❍	❍

d. Other, please specify: ____

11. In the last 4 weeks (i.e. 1 month), how many times have you visited recreational parks in Ipoh?

________________ (times) in last 1 month

12. Please indicate 3 main transportation modes for you to get around on a regular basis for things like shopping, visiting doctors or going to other places.

1. ______________________ 2. ______________________ 3. ______________________

13. In general, how would you rate your health?

❍ Healthy ❍ Inactive / with restricted mobility

❍ Active but with underlying diseases (DM, HPT, others) ❍ Hospitalised / bed-ridden

14. Do you have any kinds of health care coverage (insurance / government pensioner / military healthcare / others)?

❍ Yes, please specify: _________________________ ❍ No ❍ Not sure

15. How often do you engage in some form of physical exercise (such as walking, jogging, biking, swimming, yoga / stretching / *tai ci*, others) in a week?

❍ Frequently (5-7times) ❍ Seldom (1-4 times) ❍ Never

16. In general, do you go online to access the Internet?

❍ Yes, please specify the purpose: ❍ No ❍ Not familiar with smart devices or computers

____

17. How do you interact with your friends, family or neighbors in your community? (example: via phone, email, face-to-face, social media, etc.)

____

18. Do you involve in any continuing education or self-improvement classes/workshops/programs in your community?

❍ Yes, please specify: ________________________ ❍ No ❍ Not sure

19. Which of the following best describes your current employment status?

❍ Self-employed (including babysitting grandchildren) ❍ Retired / pensioner

❍ Employed ❍ Unemployed

20. What is/are your source(s) of income?

❐ Own salary (still working) ❐ Pension ❐ None

❐ Welfare / other government or non-profit organization, please specify: ____________________________

❐ Family members (spouse / children / grandchildren / relatives)

❐ Others, please specify: __________________

21. What is your average income per month (if any)? (RM) __________________

22. Do you wish to continue to work for as long as possible?

❍ Yes ❍ No ❍ Not sure

23. Does Ipoh have the following? How important do you think it is to have the following in Ipoh?

	(Based on IPOH city)	Availability			Importance		
		Yes	No	Not sure	Important	Not important	Not sure
H1	Homes that are equipped with features such as a no-step entry, wider doorways, first floor bedroom and bath, grab bars in bathrooms	❍	❍	❍	❍	❍	❍
H2	Affordable housing options for senior people	❍	❍	❍	❍	❍	❍

	(Based on IPOH city)	Availability			Importance		
		Yes	No	Not sure	Important	Not important	Not sure
H3	Financial assistance for home modification / purchasing	❍	❍	❍	❍	❍	❍
O4	Accessibility of the following to older people:						
	i) Park and recreational areas	❍	❍	❍	❍	❍	❍
	ii) Public building and facilities (eg. hospital, post office, bank, offices, telephone booth, others)	❍	❍	❍	❍	❍	❍
	iii) Public rest rooms accessible to older people of different physical abilities	❍	❍	❍	❍	❍	❍
O5	Well-maintained parks, public building and facilities	❍	❍	❍	❍	❍	❍
O6	Neighborhood watch programs (eg: *RELA, Rukun Tetangga*)	❍	❍	❍	❍	❍	❍
T7	Accessible and convenient public transportation	❍	❍	❍	❍	❍	❍
T8	Affordable public transportation	❍	❍	❍	❍	❍	❍
T9	Public transport provide access to destinations like hospital, clinics, parks, shopping centers, banks and other key destination.	❍	❍	❍	❍	❍	❍
T10	Easy to read traffic signs (eg. appropriate size, colour and font)	❍	❍	❍	❍	❍	❍
T11	Priority parking bays for elderly	❍	❍	❍	❍	❍	❍
T12	Audio / visual pedestrian crossings	❍	❍	❍	❍	❍	❍

	(Based on IPOH city)	Availability			Importance		
		**Yes**	**No**	**Not sure**	**Important**	**Not important**	**Not sure**
H13	Health and wellness programs and classes in areas such as nutrition, smoking cessation, and weight control	❍	❍	❍	❍	❍	❍
H14	Conveniently located health facilities	❍	❍	❍	❍	❍	❍
H15	Home visit services by healthcare professionals for senior patients	❍	❍	❍	❍	❍	❍
H16	Nursing home for older people	❍	❍	❍	❍	❍	❍
H17	A variety of health care professionals including specialists	❍	❍	❍	❍	❍	❍
H18	Health care professionals who speak different languages	❍	❍	❍	❍	❍	❍
H19	Respectful and helpful hospital and clinic staff	❍	❍	❍	❍	❍	❍
S20	Privilege for senior citizens for entertainment (eg. discount, special program, no queue, etc.)	❍	❍	❍	❍	❍	❍
S21	Conveniently located venues for entertainment	❍	❍	❍	❍	❍	❍
S22	A variety of cultural celebration (festivals, spiritual events, etc.) involving older adults in the multiracial populations	❍	❍	❍	❍	❍	❍
S23	Social clubs for books, gardening, crafts or hobbies	❍	❍	❍	❍	❍	❍
V24	A range of volunteer activities to choose from for elderly	❍	❍	❍	❍	❍	❍

	(Based on IPOH city)	Availability			Importance		
		Yes	No	Not sure	Important	Not important	Not sure
V25	Opportunities for older adults to participate in decision making bodies such as community councils or committees (eg. giving expert opinion)	❍	❍	❍	❍	❍	❍
J26	A range of flexible job opportunities for older adults (eg. part-time)	❍	❍	❍	❍	❍	❍
C27	Official, written information (e.g.: forms, brochures) adapted to the needs of seniors (e.g.: large font size)	❍	❍	❍	❍	❍	❍
C28	Telephone operator services adapted to the needs of seniors (e.g.: instructions are given slowly)	❍	❍	❍	❍	❍	❍
C29	Free access to computers and the Internet in public places such as the library, centers or government buildings	❍	❍	❍	❍	❍	❍
C30	Information (eg. flyers, maps, others) that is available in a number of different languages	❍	❍	❍	❍	❍	❍

24. Overall, do you think Ipoh is an age-friendly city?

❍ Yes ❍ No ❍ Not sure

25. Can the study team contact you for further information if required in future on the Age-friendly city concept in Ipoh?

❍ Yes (proceed with the Informed Consent for contact details) ❍ No

The research team would like to express sincere gratitude for your time and effort in contributing for this study.

Thank you.
